# Technology Acceptance for an Intelligent Comprehensive Interactive Care (ICIC) System for Care of the Elderly: A Survey-Questionnaire Study

**DOI:** 10.1371/journal.pone.0040591

**Published:** 2012-08-01

**Authors:** Alice M. K. Wong, Wei-Han Chang, Pei-Chih Ke, Chun-Kai Huang, Tsai-Hsuan Tsai, Hsien-Tsung Chang, Wann-Yun Shieh, Hsiao-Lung Chan, Chih-Kuang Chen, Yu-Cheng Pei

**Affiliations:** 1 Department of Physical Medicine and Rehabilitation, Chang Gung Memorial Hospital at Taoyuan, Taoyuan, Taiwan; 2 Graduate Institute of Rehabilitation Science, Chang Gung University, Taoyuan, Taiwan; 3 Healthy Aging Research Center, Chang Gung University, Taoyuan, Taiwan; Federal University of Rio de Janeiro, Brazil

## Abstract

The key components of caring for the elderly are diet, living, transportation, education, and safety issues, and telemedical systems can offer great assistance. Through the integration of personal to community information technology platforms, we have developed a new Intelligent Comprehensive Interactive Care (ICIC) system to provide comprehensive services for elderly care. The ICIC system consists of six items, including medical care (physiological measuring system, *Medication Reminder*, and *Dr. Ubiquitous*), diet, living, transportation, education (*Intelligent Watch*), entertainment (*Sharetouch*), and safety (*Fall Detection*). In this study, we specifically evaluated the users' intention of using the *Medication Reminder*, *Dr. Ubiquitous*, *Sharetouch*, and *Intelligent Watch* using a modified technological acceptance model (TAM). A total of 121 elderly subjects (48 males and 73 females) were recruited. The modified TAM questionnaires were collected after they had used these products. For most of the ICIC units, the elderly subjects revealed great willingness and/or satisfaction in using this system. The elderly users of the *Intelligent Watch* showed the greatest willingness and satisfaction, while the elderly users of *Dr. Ubiquitous* revealed fair willingness in the dimension of perceived ease of use. The old-old age group revealed greater satisfaction in the dimension of result demonstrability for the users of the *Medication Reminder* as compared to the young-old and oldest-old age groups. The women revealed greater satisfaction in the dimension of perceived ease of use for the users of *Dr. Ubiquitous* as compared to the men. There were no statistically significant differences in terms of gender, age, and education level in the other dimensions. The modified TAM showed its effectiveness in evaluating the acceptance and characteristics of technologic products for the elderly user. The ICIC system offers a user-friendly solution in telemedical care and improves the quality of care for the elderly.

## Introduction

In Taiwan and around the world, aging and increasing longevity have been having an impact on many aspects of daily life. In combination with the decreased fertility rate, improving medical standards have increased the elderly population, making Taiwan a rapidly aging society [1]. More and more elderly individuals are either living alone or are home alone during the day because their family members are out working [2]. Proper care systems, such as long-distance telemedical care, must be established in order to offer appropriate and alternative care options for the elderly population.

Telemedical care, however, services only one aspect of the elderly's needs. Diet, living, transportation, education, and safety issues are also important components of elderly care that should not be ignored. Therefore, we have developed a new Intelligent Comprehensive Interactive Care (ICIC) system to provide comprehensive services for care of the elderly through the integration of personal to community information technology (IT) platforms. The ICIC system is comprised of six service items: (1) medical care (including a physiological measuring system, medication reminder system, and doctor-patient interactive system), (2) diet, living, transportation, and education (which were integrated into the medical care system by an *Intelligent Watch*), (3) entertainment (*Sharetouch*), and (4) safety (*Fall Detection*). However, we were uncertain whether the elderly would accept and/or be satisfied with these latest technologies and services.

Since the elderly need to be more certain before they act, they are usually among the last to adopt a product, service, or innovative idea. In addition, the elderly tend to have relatively negative views toward technology and show less interest in using various new technologies [3]. It has been shown that the computer-using experience, perceived ease of use, perceived usefulness, self-attitude toward new technology, and socialization agents could predict the acceptance of technology [4]–[9]. For the elderly population, it is also important to understand the factors affecting their adoption of technology during an assessment of technology acceptance.

The technological acceptance model (TAM) has been widely applied in many IT research fields [01]–[12]. The model is superior in its strong explanatory ability for evaluating users' behavioral intentions around using IT products [13] and novel communication technologies [12]. A modified version of TAM (TAM-2) [14], [15] that includes social (*e.g.,* subjective norm) and cognitive influences (*e.g.,* job relevance and perceived ease of use), can be applied to achieve more reliable evaluation outcomes.

In this study, we evaluated the users' intentions and satisfaction level specifically in four units of the new ICIC system (*Medication Reminder*, *Doctor-Patient Interactive System*, *Sharetouch,* and *Intelligent Watch*) using TAM-2. We hypothesized that these products can offer user-friendly solutions for long-distance telemedical care and the monitoring of elderly subjects. In addition, these products were expected to improve social life and quality of care in the elderly population.

## Methods

### Subjects

We recruited 121 subjects (48 men and 73 women) from Chang Gung University, Chang Gung Memorial Hospital, and Chang Gung Silver Village for the elderly between July 2009 and June 2010. They were either elderly community residents who lived independently (n = 92), or caregivers taking care of patients in the rehabilitation wards (n = 29). The distribution of subjects by demographical data in each subcategory is shown in Table 1. Subjects with a history of psychological problems, mental and motor dysfunctions, or cognitive deficits were excluded from this study. We obtained ethics approval for our study from the Institutional Review Board (the “IRB”) of Chang Gung Medical Foundation. All relevant ethical safeguards have been met in relation to patient or subject protection. After we meticulously explained the purposes of this study to the subjects, all subjects signed informed consent forms before participating.

**Table 1:** Demographic data of the subjects who received TAM-2 evaluation for each ICIC unit.

	Gender			
Subcategory	Male	Female	Age (year ± SD)	Age range
Medication Reminder	7	7	79.3±6.1	71–88
Dr. U	14	15	68.5±4.8	65–84
Sharetouch	17	32	78.3±7.5	65–91
Intelligent Watch	10	19	55.9±6.7	41–72

## Materials

Here are short explanations of the design and function of each of the ICIC units we evaluated using TAM-2:

### Medication Reminder

The *Medication Reminder* was integrated using sensor-detected and internet-based techniques. This system reminds the patients to take medicine on time according to the physician's prescriptions, and sends crucial information on the medication to their family members via the internet. The main function of *Medication Reminder* is to decrease the chances of taking medications with the wrong dosage or at the wrong time. Subjects operated the system using their own accounts, which provided information on the timing and dosage of the medication. A radio frequency identification (RFID) tag was attached to the drug bag and, as a result, the system would accurately register the removal whenever the subjects took medications from the drug bag. If the subjects did not take out the drugs correctly, the system would immediately alert their family members via the internet. Family members can be notified through the internet choices of electronic mail and Windows Messenger (MSN), or via cell phone, etc. A total of 18 elderly residents (9 men and 9 women) living in the Chang Gung Silver Village participated in using the *Medication Reminder*. Personnel acquainted with the system taught these elderly subjects to ensure that they learned to properly use the system.

### Doctor-Patient Interactive System

The *Doctor-Patient Interactive System*, also called *Dr. Ubiquitous* (*Dr. U*), is a personal and multifunctional tool for consultation on daily health problems. *Dr. U* consults on clinical symptoms, measures physiological data, and provides basic health information. This system provides users with crucial suggestions and answers to the health problems they are facing. Other non-medical treatment information, such as suggestions for implementing the proper exercises to improve certain health conditions, is also offered. A total of 30 elderly residents (14 men and 16 women) living in Chang Gung Silver Village participated in the use of *Dr. U*. Personnel acquainted with the system taught these elderly subjects to ensure that they learned to use the system properly.

### Sharetouch

**Figure 1:**
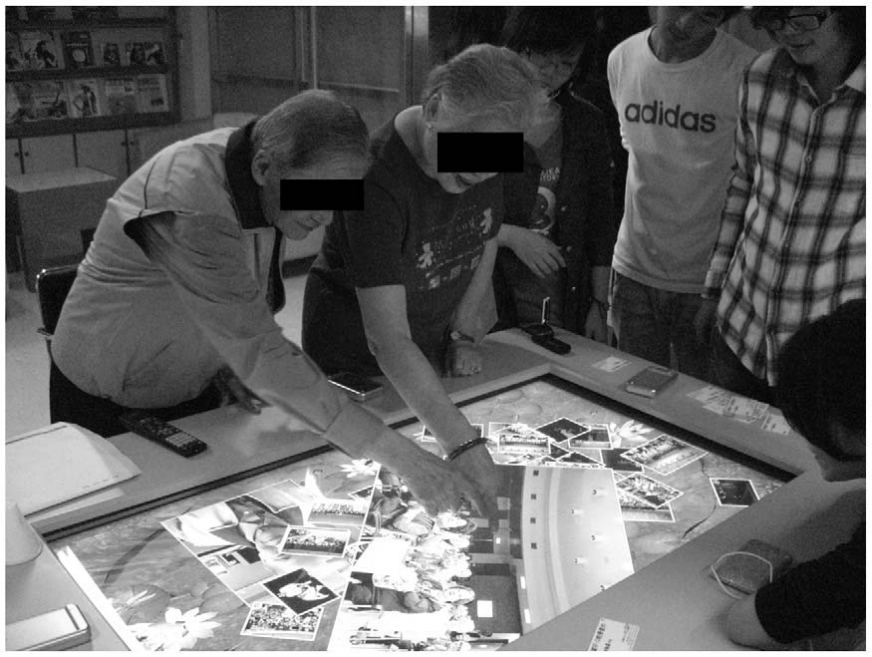
Demonstration of the interactive use of *Sharetouch* by two elderly subjects.

*Sharetouch* is an entertainment system specifically designed for the elderly community. It offers communication, data (photo, video, music, and document) sharing, and interactive game functions (Figure 1). *Sharetouch* is an all-in-one computer system with a large flat panel touch screen integrated on a desk table. It is user friendly, and two to three or even more elderly residents can gather together to use the system to share their family photos and even play interactive games with each other. Using *Sharetouch* can improve the social life of elderly residents. A total of 52 elderly residents (18 men and 34 women) living in the Chang Gung Silver Village participated in the evaluation of *Sharetouch*. The basic entertainment and communication functions of *Sharetouch* were patiently taught to these elderly subjects to ensure they were comfortable using the system.

### Intelligent Watch

The *Intelligent Watch* was designed for real-time monitoring of the subjects' physiological conditions. It consisted of an electrocardiogram (EKG), time display, and notification of events (such as weather predictions and medication reminders). The Watch measured EKG data and computed the subjects' heart rate from the EKG signals. A secure digital memory card (SD card) was integrated into the Watch to store the heart rate and EKG data. The weather prediction information was included to assist the elderly subjects in choosing the proper attire before participating in outdoor events. A total of 30 caregivers (10 men and 20 women) taking care of patients in the Chang Gung Memorial Hospital rehabilitation wards participated in using the *Intelligent Watch*. Personnel acquainted with the system demonstrated the proper of use of the *Intelligent Watch* to ensure that the caregivers could use the Watch properly.

### Questionnaire Design

The TAM-2 questionnaire includes ten dimensions and was used in this study to construct the questionnaires for evaluating the ICIC system. One part of the questionnaire evaluates the willingness to use technologic products, such as behavior intention and result demonstrability, and another part evaluates satisfaction, such as the perceived usefulness, perceived ease of use, and output quality. The questionnaire was translated into Chinese with scores ranging from one to seven in TAM-2 for *Medication Reminder*, *Sharetouch*, and *Intelligent Watch* and from one to five in TAM-2 for *Dr. U*. The higher the score, the higher the intention or the greater the satisfaction was in using the ICIC system. In the seven-grade system, scores of one and two indicated poor willingness or satisfaction, scores of three and four indicated fair willingness or satisfaction, scores of five and six indicated good willingness or satisfaction, and a score of seven indicated excellent willingness or satisfaction. In the five-grade system, scores of one and two indicated poor willingness or satisfaction, a score of three indicated fair willingness or satisfaction, a score of four indicated good willingness or satisfaction, and a score of five indicated excellent willingness or satisfaction. Each questionnaire can be divided into several subcategories. Considering that the TAM-2 questionnaire is designed for the elderly, we selected several dimensions, such as “Behavior intention”, “Perceived usefulness”, “Perceived ease of use”, “Output quality”, and “Result demonstrability” according to the characteristics of the ICIC units. In the evaluation of *Dr. U* which has interesting and lively suggestions and answers to the health problems and *Sharetouch*, “Enjoyment” was included in the questionnaire because its major goal includes the effect of entertainment.

### Statistical Analysis

We used SPSS 10.0 (Chicago, Illinois) for statistical analysis. We first performed descriptive analysis for the demographic data and the results of the TAM-2 questionnaires. The TAM-2 scores in *Dr. U* used a 5-level scoring system, so we performed a normalization process to transfer the score into the 7-level scoring system so that the data could be compared among the ICIC units. We used the Friedman test to compare the differences in the TAM-2 scores within each ICIC unit and the Kruskal-Wallis test to compare the differences in the scores across the ICIC units for the same question in TAM-2. We used the Mann-Whitney test to compare the differences in the TAM-2 scores between the gender subgroups and the Kruskal-Wallis test to compare the differences among the age subgroups or education level subgroups. Data were expressed in terms of mean and standard deviation. Statistical significance was set at a *p* value of 0.05.

## Results

The questions in all the questionnaires and the distribution of TAM-2 scores among all the participants are shown in Supplementary Information (Tables S1, S2, S3, and S4). The comparison of the TAM-2 scores among the TAM-2 dimensions showed that the TAM-2 scores differed significantly among the dimensions in *Dr. U* (*p*<0.001), *Sharetouch* (*p*<0.001), and *Intelligent Watch* (*p*<0.001), but not in *Medication Reminder* (*p* = 0.66) (Table 2). Specifically, *Dr. U* had a high score in perceived usefulness (5.8±0.8) and a low score in perceived ease of use (4.4±0.7). *Sharetouch* had a high score in output quality (6.0±0.9) and a low score in perceived ease of use (5.3±1.1). *Intelligent Watch* had a high score in behavior intention (6.5±0.9) and a low score in output quality (5.3±1.4). To summarize, the TAM-2 demonstrated the strengths and limitations of each ICIC unit.

**Table 2:** Comparison of the normalized TMA-2 scores (mean score) among ICIC units and among TAM-2 dimensions.

	BI	PU	PEU	OQ	RD	En	*p* value
Medication Reminder	5.1±2.2	5.6±1.4	5.4±1.5	5.3±1.7	5.8±1.3		0.66
Dr. U	5.3±1.2	5.8±0.8	4.4±0.7	5.2±0.6	5.6±0.9	5.4±1.3	<0.001***
Sharetouch	5.7±1.1	5.5±1.1	5.3±1.1	6.0±0.9	5.4±1.2	5.4±1.0	<0.001***
Intelligent Watch	6.5±0.9	6.2±1.0	6.1±0.8	5.3±1.4	6.5±0.6		<0.001***
*p* value	<0.001***	0.06	<0.001***	0.02*	<0.001***	0.97	

* : *p*<0.05; **: *p*<0.01, ***: *p*<0.001.

Abbreviations: BI  =  behavior intention, PU  =  perceived usefulness, PEU  =  perceived ease of use, OQ  =  output quality, RD  =  result demonstrability, En  =  enjoyment.

For most dimensions in TAM-2, we observed a statistically significant difference among the ICIC units. Specifically, the TAM-2 scores differed among the ICIC units in behavior intention (*p*<0.001), perceived ease of use (*p*<0.001), output quality (*p* = 0.02), and result demonstrability (*p*<0.001). For behavior intention, the highest score occurred in *Intelligent Watch* (6.5±0.9), and the lowest score in *Medication Reminder* (5.1±2.2). For perceived ease of use, the highest score was found in *Intelligent Watch* (6.1±0.8) and the lowest score in *Dr. U* (4.4±0.7). For output quality, the highest score was observed in *Sharetouch* (6.0±0.9) and the lowest score in *Dr. U* (5.2±0.6). For result demonstrability, the highest score was reported in *Intelligent Watch* (6.5±0.6) and the lowest score in *Sharetouch* (5.4±1.2). There was a borderline significant difference (*p* = 0.06) among the different ICIC units in perceived usefulness, but the scores in this dimension all revealed good satisfaction (5.5–6.2). In the dimension of enjoyment, only *Dr. U* and *Sharetouch* were compared. The results showed that the enjoyment score in these two ICIC units was comparable (*p* = 0.97).

### Medication Reminder

For *Medication Reminder*, the results indicated that most of the elderly participants expressed great willingness (5.1±2.2 in behavior intention and 5.8±1.3 in result demonstrability) and good satisfaction (5.6±1.4 in perceived usefulness, 5.4±1.5 in perceived ease of use, and 5.3±1.7 in output quality) in using this system (Table 2).

In terms of gender, the men revealed greater willingness and satisfaction than the women in using *Medication Reminder* (Table 3), but there was no statistical significance between the male and female subgroups. In terms of age, the middle-old age group (ages 75–84) revealed greater willingness and satisfaction in using the *Medication Reminder* as compared to their young-old (ages 65–74) and oldest-old counterparts (age 85 and older). In result demonstrability, there was statistical significance between the young-old age, old-old age, and oldest-old age groups (*p* = 0.02).

**Table 3:** Gender and age differences in the TAM-2 scores (mean score) for *Medication Reminder* (n = 14).

	Gender			Age			
	Male	Female		Young-old	Old-old	Oldest-old	
	n = 7	n = 7	*p* value	n = 4	n = 7	n = 3	*p* value
BI	5.7±2.1	4.6±2.4	0.29	5.1±1.3	5.3±2.6	4.8±2.9	0.80
PU	5.7±1.7	5.6±1.3	0.84	5.0±1.4	6.2±1.1	5.2±2.0	0.38
PEU	5.5±1.5	5.2±1.6	0.60	5.0±1.7	5.9±1.5	4.7±1.0	0.25
OQ	5.5±1.6	5.1±1.8	0.61	4.3±1.7	5.9±1.8	5.2±0.6	0.09
RD	5.9±1.3	5.7±1.3	0.79	4.4±0.7	6.7±0.5	5.4±1.5	0.02*
O.M.	5.7±1.3	5.2±1.0	0.52	4.8±1.1	6.0±1.1	5.1±0.5	0.14

* : *p*<0.05.

Abbreviations: BI  =  behavior intention, PU  =  perceived usefulness, PEU  =  perceived ease of use, OQ  =  output quality, RD  =  result demonstrability, O.M.  =  overall mean.

### Dr. U

In *Dr. U*, most of the elderly participants revealed good willingness (5.3±1.2 in behavior intention and 5.6±0.9 in result demonstrability) and satisfaction (5.8±0.8 in perceived usefulness and 5.2±0.6 in output quality) in using this system, but only fair satisfaction (4.4±0.7) in the dimension of perceived ease of use (Table 2). In terms of the gender subgroups, the women showed greater satisfaction than the men, as evidenced by the statistical significance difference in perceived ease of use (*p* = 0.04) (Table 4). In terms of age, we found no statistical significance in the TAM-2 scores between the age subgroups (*p*>0.05). Similarly in terms of education, we found no statistical significance in scores between the education subgroups (*p*>0.05).

**Table 4:** Gender, age, and education differences in the TAM-2 scores (mean scores) for *Dr. U* (n = 29).

	Gender			Age			Education				
	Male	Female		Y–O	O–O & Oest-O		PS	JS	SHS	B	
	n = 14	n = 15	*p* value	n = 24	n = 5	*p* value	n = 2	n = 12	n = 3	n = 12	*p* value
BI	5.2±1.2	5.3±1.3	0.64	5.3±1.2	5.4±1.7	0.88	5.2±1.0	5.2±1.5	5.0±0.9	5.4±1.2	0.94
PU	5.9±0.9	5.7±0.8	0.79	5.9±0.8	5.6±1.1	0.38	5.8±0.5	5.7±0.9	5.6±0.3	6.1±0.9	0.68
PEU	4.2±0.7	4.7±0.7	0.04*	4.5±0.8	4.3±0.7	0.90	5.1±1.6	4.6±0.7	4.8±0.8	4.1±0.5	0.22
OQ	5.2±0.6	5.4±0.6	0.11	5.3±0.6	5.1±0.8	0.86	5.4±0.5	5.5±0.6	5.0±0.7	5.0±0.7	0.59
RD	5.7±1.0	5.6±1.0	0.95	5.7±0.9	5.4±1.2	0.41	5.1±1.0	5.6±1.0	5.3±0.4	5.9±0.9	0.67
En	5.3±1.4	5.4±1.2	0.87	5.4±1.3	5.2±1.4	0.61	4.4±0.5	5.3±1.3	5.5±1.5	5.6±1.4	0.49
O.M.	5.2±0.7	5.3±0.7	0.54	5.3±0.6	5.2±1.0	0.77	5.2±0.9	5.3±0.8	5.2±0.5	5.3±0.7	0.95

* : *p*<0.05.

Abbreviations: Y–O  =  young-old, O–O  =  old-old, Oest-O  =  oldest-old, PS  =  primary school, JS  =  junior school, SHS  =  senior high school, B  =  Bachelor, BI  =  behavior intention, PU  =  perceived usefulness, PEU  =  perceived ease of use, OQ  =  output quality, RD  =  result demonstrability, En  =  enjoyment, O.M.  =  overall mean.

### Sharetouch

Most of the elderly participants revealed good willingness and satisfaction in the use of this system (Table 2). In terms of gender, age, or education, we noticed no statistically significant differences when we compared the subgroups (*p*>0.05) (Table 5).

**Table 5:** Gender, age, and education differences in the TAM-2 scores (mean score) for *Sharetouch* (n = 49).

	Gender			Age				Education				
	Male	Female		Y–O	O–O	Oest-O		PS	JS	SHS	B	
	n = 17	n = 32	*p* value	n = 11	n = 29	n = 9	*p* value	n = 5	n = 7	n = 14	n = 23	*p* value
BI	5.9±1.1	5.6±1.1	0.34	5.7±0.8	5.8±1.3	5.3±0.8	0.13	6.6±0.4	5.7±1.1	5.6±1.3	5.6±1.0	0.15
PU	5.5±1.0	5.5±1.1	0.84	5.7±1.0	5.5±1.2	5.3±0.8	0.44	6.1±0.6	6.0±0.8	5.4±1.4	5.3±1.0	0.39
PEU	5.3±1.1	5.1±1.0	0.50	5.2±0.9	5.3±1.2	5.3±0.8	0.78	5.7±0.9	5.8±0.7	4.9±1.4	5.2±0.9	0.38
OQ	5.9±1.0	6.1±0.9	0.72	6.0±0.9	6.0±1.0	6.3±0.6	0.83	6.3±1.0	6.2±1.1	5.8±1.1	6.0±0.8	0.69
RD	5.4±1.1	5.3±1.2	0.85	5.7±1.0	5.2±1.4	5.4±0.7	0.56	5.9±0.9	5.7±1.1	4.7±1.5	5.5±0.9	0.27
En	5.3±1.3	5.4±0.8	0.88	5.4±0.9	5.5±1.0	5.2±1.0	0.52	5.9±0.6	5.5±0.5	5.0±1.3	5.5±1.0	0.48
O.M.	5.5±0.8	5.5±0.9	0.99	5.6±0.8	5.5±1.0	5.4±0.5	0.70	6.0±0.6	5.9±0.6	5.2±1.2	5.5±0.6	0.26

Abbreviation: Y–O  =  young-old, O–O  =  old-old, Oest-O  =  oldest-old, PS  =  primary school, JS  =  junior school, SHS  =  senior high school, B  =  Bachelor, BI  =  behavior intention, PU  =  perceived usefulness, PEU  =  perceived ease of use, OQ  =  output quality, RD  =  result demonstrability, En  =  enjoyment, O.M.  =  overall mean.

### Intelligent Watch

Most of the elderly participants revealed great willingness and satisfaction in using *Intelligent Watch*, and the mean scores in most dimensions were highest among all the ICIC units (Table 2). In terms of gender, age, and education, we noticed no statistically significant differences when we compared the subgroups (*p*>0.05) (Table 6).

**Table 6:** Gender, age, and education differences in the TAM-2 scores (mean score) for *Intelligent Watch* (n = 29).

	Gender			Age (years)				Education				
	Male	Female		41–50	51–60	>61		PS	JS	SHS	B	
	n = 10	n = 19	*p* value	n = 6	n = 17	n = 6	*p* value	n = 9	n = 8	n = 7	n = 5	*p* value
BI	6.5±1.0	6.5±0.9	0.75	6.9±0.2	6.4±0.9	6.2±1.2	0.33	6.2±1.2	6.7±0.5	6.3±1.1	6.7±0.7	0.82
PU	6.2±1.1	6.1±0.9	0.74	6.8±0.3	6.0±1.0	5.9±1.2	0.13	6.0±1.0	6.4±0.9	6.2±1.0	6.0±1.1	0.81
PEU	6.1±0.8	6.1±0.8	0.93	6.3±0.7	6.1±0.7	6.0±1.0	0.94	6.2±0.9	6.3±0.7	5.9±0.7	5.9±0.6	0.36
OQ	5.0±1.8	5.5±1.2	0.54	5.8±1.2	5.3±1.6	4.8±0.8	0.30	5.5±1.3	5.4±0.9	5.0±2.1	5.3±1.4	0.99
RD	6.4±0.7	6.5±0.6	0.98	6.6±0.5	6.3±0.7	6.8±0.5	0.29	6.6±0.6	6.5±0.5	6.4±0.5	6.2±1.1	0.95
O.M.	6.1±0.8	6.2±0.6	0.98	6.5±0.4	6.1±0.7	6.0±0.7	0.42	6.1±0.8	6.3±0.5	6.0±0.8	6.0±0.8	0.90

Abbreviations: PS  =  primary school, JS  =  junior school, SHS  =  senior high school, B  =  Bachelor, BI  =  behavior intention, PU  =  perceived usefulness, PEU  =  perceived ease of use, OQ  =  output quality, RD  =  result demonstrability, O.M.  =  overall mean.

## Discussion

For more than 25 years, the adoption of new technologies by the elderly has been the focus of study [12]. During this time, the factors affecting the elderly adoption of new technologies included ease of use, cost, poor trial use, a higher score on innovation, training, income, and education. Mathur (1999) also indicated that socialization agents (the influence of young family members) affected the adoption of technologies [8]. Among these factors, the ease of use and cost of technologies, the attitude of elderly individuals toward technology, and the social agents are modifiable factors. Thus, in the present study, we developed the ICIC system with the concepts of usefulness and ease of use, and the intention of enabling elderly interaction with family, friends, and medical staffs through an IT platform.

TAM has been widely applied to many areas of information technology. This model provides a theoretical basis for understanding the internal beliefs, attitudes, and intentions of the elderly in using IT products [16], [17]. For elderly people, their intrinsic motivation would affect their perception on ease of use and perceived enjoyment in using IT products [15], [18], [19]. In the present study, we applied the modified version of TAM, the TAM-2, [14], [15] that includes the dimensions of behavior intention (*e.g.,* behavior intention) and cognitive instrumental (*e.g.,* output quality and perceived ease of use) in order to achieve a comprehensive assessment. The TAM-2 questionnaires in the present study were translated into a Chinese version checked for validity by expert scholars in a clinical trial meeting of *Intelligent Watch* [20].

The elderly subjects in this study were recruited from a retirement village, where computer courses are regularly taught to the elderly residents. In order to prevent experimental bias, our evaluations were carried out immediately after the elderly subjects were taught how to use the ICIC system. If the evaluations had been carried out after repeated use of the ICIC system, results such as ease of use would have been greatly affected and would have then become unreliable. The results in all dimensions were almost always at least “good” in each of the ICIC units, except for the “perceived ease of use” in *Dr. U*. Caregivers using the *Intelligent Watch* revealed the greatest willingness and satisfaction in most dimensions, as compared to the other ICIC units. Most of the ICIC units except for *Medication Reminder* had significant differences in the results between each dimension. These revealed that each single ICIC unit has its own advantages and limitations for elderly users, resulting in score discrepancies among the dimensions within TAM2 and indicating the validity of TAM2 for elderly users. Most dimensions except “perceived usefulness” and “enjoyment” differed across the ICIC units, indicating that there were different characteristics across the different ICIC units and that TAM-2 effectively illustrated those differences. According to our results, we identified the attitude of the elderly toward using these technological products, and this knowledge will guide us in modifying and improving the present project to ensure better user acceptance among the elderly population.

As for the factors of gender, age, and education influencing the acceptance of technology for elderly, we only found a significant difference in the result of demonstrability between the young-old, old-old, and oldest-old age groups for the use of *Medication Reminder*, in which the elderly of the old-old age subgroup showed the greatest acceptance. We also only noticed a significant difference in “perceived usefulness” between the men and women using *Dr. U*, where the elderly women indicated greater satisfaction than the men. Most dimensions in TAM-2 did not reveal significant differences between the subgroups of gender, age, or education in each of the ICIC units, a finding that is not compatible with results reported earlier [21], [22]. This discrepancy might be explained by the relatively small sample size and the choice of a relatively homogenous sample recruited for the present study.

Aging is a continuous and highly complex process that presents changes in two main categories: biophysical and psychosocial [12]. We designed the ICIC system to assist the elderly in coping with the biophysical aspect of aging. In contrast, the psychosocial or mental aspects of aging were not adequately explored in the present study design. The psychosocial side of aging is related to the development of the disorientation and dementia observed in old age [23]. The question remains whether IT products could be effective in assisting with the psychosocial side of aging. Further research is mandatory to address this question.

In the development of ICIC system, intelligent control of interconnected systems is an important issue. To understand how interconnected systems behave involves fundamental theories in modeling, optimization, and control. The interconnected systems demonstrate complex, nonlinear and multiple time-delay behaviors that composed of a number of interdependent subsystems [24], [25]. Recent progresses in interconnected systems have led advancements in artificial intelligence in methodology (such as Lyapunov method [26]–[29], Takagi-Sugeno fuzzy model and parallel-distributed compensation scheme [24], [30]) and tools (such as robustness design of fuzzy controller [29], [31]–[33], neural networks approach [34], [35] and adaptive fuzzy sliding model controller [36]) that help analyze and stabilize the nonlinear multiple time-delay interconnected systems. The aforementioned artificial intelligence methods have already successfully applied in various inter-connected systems, such as lead rubber bearing isolation hybrid protective system [37], oceanic structure and environment [38]–[40], and ecosystems [41], [42]. The ICIC system delineated in the present study may exemplify an application of interconnected systems. Recent advance in artificial intelligence has made the information-based elder care possible. We hypothesize that the free flow of information among elderly users and between medical providers may enhance our quality of care. The development of ICIC system supplemented by these novel artificial intelligences may provide us reliable database and home-based intelligence devices that will change the way we care the elderly in the near future.

The present study does have shortcomings. The sample size was relatively small. Although most of the recruited elderly subjects showed good willingness and satisfaction with our ICIC products, further investigation is needed to answer whether these products can substantially improve the subjects' quality of life and the activities of daily living. In the next stage of study, a larger sample size of elderly subjects will be taught to wear the *Intelligent Watch* as we monitor the temporal changes in their functional status prior to and following the use of these technologies.

The elderly population showed good technology acceptance for our newly developed ICIC system that provides a feasible interdisciplinary model integrating medical care, diet, living, transportation, education, entertainment, and safety demands. We report here on the first of a series of studies we have conducted to test the willingness and satisfaction of elderly subjects using this system, and we have demonstrated at least a fair-to-good acceptance in this population. The system offers a user-friendly solution in telemedical care and monitoring and can improve social interaction and quality of care for the elderly population.

## Supporting Information

Table S1 **Distribution TAM-2 scores for** ***Medication Reminder*** **(n = 14).**
(DOC)

Table S2 **Distribution TAM-2 scores for** ***Dr.U*** **(n = 29).**
(DOC)

Table S3 **Distribution TAM-2 scores for** ***Sharetouch*** **(n = 49).**
(DOC)

Table S4 **Distribution TAM-2 scores for** ***Intelligent Watch*** **(n = 29).**
(DOC)
