# Supplementary material for: Technology Acceptance for an Intelligent Comprehensive Interactive Care (ICIC) System for Care of the Elderly: A Survey-Questionnaire Study
Source: PLoS One. 2012 Aug 1;7(8):e40591. doi: 10.1371/journal.pone.0040591 (PMC3411612; doi:10.1371/journal.pone.0040591)
Supplement: Table S1 — Distribution TAM-2 scores for Medication Reminder (n = 14). (DOC) [file pone.0040591.s001.doc]

| **Table S1.** Distribution TAM-2 scores for *Medication Reminder* (n=14) | | | | | | | | |
| --- | --- | --- | --- | --- | --- | --- | --- | --- |
| Item | Distribution in percentage (%) | | | | | | | Mean score |
|  | Excellent | Good | | Fair | | Poor | |  |
|  | 7 | 6 | 5 | 4 | 3 | 2 | 1 |  |
| 1. If I got a chance, I would use “Medication Reminder.” | 50 | 5.6 | 11.1 | 11.1 | 5.6 | 5.6 | 11.1 | 5.2±2.2 |
| 2. If gave me “Medication Reminder,” I would definitely use it. | 50 | 6.3 | 12.5 | 6.3 | 0 | 0 | 25 | 5.0±2.6 |
| 3. Using “Medication Reminder” improves the quality of taking care of me. | 43.8 | 12.5 | 12.5 | 6.3 | 6.3 | 6.3 | 12.5 | 5.1±2.3 |
| 4. Using “Medication Reminder” enhances my ability on taking care of me. | 43.8 | 12.5 | 12.5 | 0 | 12.5 | 6.3 | 12.5 | 5.1±2.3 |
| 5. Using “Medication Reminder” saves me time, and makes it easier to take care of me. | 56.3 | 18.8 | 12.5 | 0 | 6.3 | 0 | 6.3 | 5.9±1.7 |
| 6. Using “Medication Reminder” improves my health. | 56.3 | 18.8 | 12.5 | 6.3 | 0 | 0 | 6.3 | 6.0±1.6 |
| 7. My interaction with “Medication Reminder” is easy for me to understand. | 31.3 | 12.5 | 6.3 | 18.8 | 18.8 | 6.3 | 6.3 | 4.8±2.0 |
| 8. I find it is easy to learn using “Medication Reminder.” | 37.5 | 6.3 | 25 | 6.3 | 6.3 | 12.5 | 6.3 | 5.0±2.1 |
| 9. Overall, I find the “Medication Reminder” easy to use. | 50 | 6.3 | 18.8 | 6.3 | 0 | 12.5 | 6.3 | 5.4±2.1 |
| 10. I find it easy to get “Medication Reminder” to do what I want it to do. | 42.9 | 14.3 | 14.3 | 21.4 | 0 | 0 | 7.1 | 5.5±1.8 |
| 11. Overall, I am satisfied with the quality of “Medication Reminder.” | 33.3 | 33.3 | 6.7 | 6.7 | 0 | 13.3 | 6.7 | 5.3±2.1 |
| 12. I have no doubt about the quality of “Medication Reminder.” | 31.3 | 12.5 | 18.8 | 12.5 | 6.3 | 12.5 | 6.3 | 4.9±2.0 |
| 13. I am glad to share the benefits of “Medication Reminder” with others. | 46.7 | 13.3 | 20 | 13.3 | 0 | 0 | 6.7 | 5.7±1.7 |
| 14. I will exchange the experience of using “Medication Reminder” with others. | 40 | 13.3 | 20 | 20 | 0 | 0 | 6.7 | 5.5±1.7 |
| 15. Obviously, using “Medication Reminder” helps me take care of me. | 33.3 | 20 | 6.7 | 20 | 6.7 | 6.7 | 6.7 | 5.1±2.0- |
| 16. I find it hard to distinguish between advantages and disadvantages. | 20 | 6.7 | 6.7 | 26.7 | 6.7 | 6.7 | 26.7 | 3.8±2.3 |
|  | | | | | | | | |
