# Supplementary material for: Technology Acceptance for an Intelligent Comprehensive Interactive Care (ICIC) System for Care of the Elderly: A Survey-Questionnaire Study
Source: PLoS One. 2012 Aug 1;7(8):e40591. doi: 10.1371/journal.pone.0040591 (PMC3411612; doi:10.1371/journal.pone.0040591)
Supplement: Table S2 — Distribution TAM-2 scores for Dr.U (n = 29). (DOC) [file pone.0040591.s002.doc]

| **Table S2.** Distribution TAM-2 scores for *Dr .U* (n=29) | | | | | | |
| --- | --- | --- | --- | --- | --- | --- |
| Item | Distribution in percentage (%) | | | | | Mean score |
|  | Excellent | Good | Fair | Poor | |  |
|  | 5 | 4 | 3 | 2 | 1 |  |
| 1. The title of “Dr. U” can draw users’ attention. | 13.3 | 50 | 26.7 | 10 | 0 | 3.7±0.0 |
| 2. The content of “Dr. U” can induce the motivation of learning or searching. | 20 | 56.7 | 10 | 13.3 | 0 | 3.8±0.0 |
| 3. The display of “Dr. U” is clear to read. | 36.7 | 53.3 | 6.7 | 3.3 | 0 | 4.2±0.0 |
| 4. “Dr. U” offers with enough information and knowledge. | 6.7 | 46.7 | 20 | 26.7 | 0 | 3.9±0.0 |
| 5. “Dr. U” offers the information with correct grammar, texts, contents, and photos. | 6.7 | 53.3 | 33.3 | 3,3 | 3.3 | 3.6±0.0 |
| 6. I can interact well with “Dr. U” actively and positively. | 33.3 | 5 | 13.3 | 3.3 | 0 | 4.1±0.0 |
| 7. “Dr. U” offers the function of interaction, practice, and feedback. | 23.3 | 56.7 | 10 | 10 | 0 | 3.9±0.0 |
| 8. The operation is easy for me to interact with “Dr. U.” (including input, output, browse, forward and backward, etc.) | 50 | 43.3 | 6.7 | 0 | 0 | 4.4±0.0 |
| 9. The photos, tables, buttons and video offered by “Dr. U” can enhance the effects of learning. | 30 | 50 | 20 | 0 | 0 | 4.1±0.0 |
| 10. The color and design in each page of internet is harmonious. | 23.3 | 53.3 | 16.7 | 6.7 | 0 | 3.9±0.0 |
| 11. It will be better if “Dr. U” adds the function of audio guidance. | 86.7 | 13.3 | 0 | 0 | 0 | 4.9±0.0 |
| 12. The duration of data display in “Dr. U” is enough. | 20 | 56.7 | 20 | 3.3 | 0 | 4.3±0.0 |
| 13. The interface of “Dr. U” corresponds to the users’ skills in computer operation. | 3.3 | 16.7 | 13.3 | 46.7 | 20 | 2.3±0.0 |
| 14. I feel the font size of “Dr. U” is proper to read. | 23.3 | 60 | 13.3 | 3.3 | 0 | 4.0±0.0 |
| 15. The arrangement of information is suitable in “Dr. U”, which classified systematically and hierarchically. | 13.3 | 46.7 | 36.7 | 3.3 | 0 | 3.7±0.0 |
| 16. “Dr. U” is available for the personal computer or other facilities (PDA). | 3.3 | 23.3 | 66.7 | 3.3 | 3.3 | 3.2±0.0 |
