# Supplementary material for: Technology Acceptance for an Intelligent Comprehensive Interactive Care (ICIC) System for Care of the Elderly: A Survey-Questionnaire Study
Source: PLoS One. 2012 Aug 1;7(8):e40591. doi: 10.1371/journal.pone.0040591 (PMC3411612; doi:10.1371/journal.pone.0040591)
Supplement: Table S3 — Distribution TAM-2 scores for Sharetouch (n = 49). (DOC) [file pone.0040591.s003.doc]

| **Table S3.** Distribution TAM-2 scores for *Sharetouch* (n=49) | | | | | | | | |
| --- | --- | --- | --- | --- | --- | --- | --- | --- |
| Item | Distribution in percentage (%) | | | | | | | Mean average |
|  | Excellent | Good | | Fair | | Poor | |  |
|  | 7 | 6 | 5 | 4 | 3 | 2 | 1 |  |
| 1. Using “Sharetouch” improves the quality of interacting with others. | 46.2 | 30.8 | 15.4 | 3.8 | 3.8 | 0 | 0 | 6.1±1.1 |
| 2. Using “Sharetouch” enhances my ability on interacting with others. | 32.7 | 21.2 | 17.3 | 23.1 | 0 | 5.8 | 0 | 5.5±1.5 |
| 3. I find “Sharetouch” helps me interact with others. | 30.8 | 25 | 15.4 | 19.2 | 3.8 | 1.9 | 3.8 | 5.4±1.6 |
| 4. Using “Sharetouch” enhances the interaction with others. | 26.9 | 25 | 19.2 | 15.4 | 3.8 | 3.8 | 5.8 | 5.2±1.7 |
| 5. My interaction with “Sharetouch” is easy for me to understand. | 21.2 | 32.7 | 23.1 | 15.4 | 0 | 7.7 | 0 | 5.4±1.4 |
| 6. I find it is easy to learn using “Sharetouch.” | 23.5 | 35.3 | 21.6 | 11.8 | 3.9 | 3.9 | 0 | 5.5±1.3 |
| 7. Overall, I find the “Sharetouch” easy to use. | 21.2 | 25 | 23.1 | 15.4 | 11.5 | 3.8 | 0 | 5.2±1.4 |
| 8. I find it easy to get “Sharetouch” to do what I want it to do. | 21.2 | 28.8 | 17.3 | 21.2 | 7.7 | 3.8 | 0 | 5.2±1.4 |
| 9. I find it will be interesting using “Sharetouch.” | 15.4 | 32.7 | 21.2 | 19.2 | 9.6 | 1.9 | 0 | 5.2±1.3 |
| 10. It is a pleasant time when using “Sharetouch.” | 15.7 | 25.5 | 23.5 | 15.7 | 9.8 | 5.9 | 3.9 | 4.9±1.6 |
| 11. I find it interesting after using “Sharetouch.” | 53.8 | 21.2 | 15.4 | 5.8 | 3.8 | 0 | 0 | 6.2±1.1 |
| 12. If I got a chance, I would use “Sharetouch.” | 51.9 | 23.1 | 17.3 | 3.8 | 3.8 | 0 | 0 | 6.2±1.1 |
| 13. If gave me “Sharetouch,” I would definitely use it. | 26.9 | 28.8 | 15.4 | 19.2 | 1.9 | 5.8 | 1.9 | 5.4±1.6 |
| 14. Overall, I am satisfied with the quality of “Sharetouch.” | 38.5 | 32.7 | 19.2 | 9.6 | 0 | 0 | 0 | 6.0±1.0 |
| 15. I have no doubt about the quality of “Sharetouch.” | 38.5 | 36.5 | 19.2 | 3.8 | 1.9 | 0 | 0 | 6.1±1.0 |
| 16. I am glad to share the benefits of “Sharetouch” with others. | 23.1 | 40.4 | 23.1 | 5.8 | 3.8 | 3.8 | 0 | 5.6±1.2 |
| 17. I will exchange the experience of using “Sharetouch” with others. | 21.2 | 28.8 | 19.2 | 17.3 | 5.8 | 7.7 | 0 | 5.2±1.5 |
| 18. I find it hard to distinguish between advantages and disadvantages. | 11.5 | 11.5 | 11.5 | 19.2 | 13.5 | 19.2 | 13.5 | 3.8±1.9 |
