# Supplementary material for: Technology Acceptance for an Intelligent Comprehensive Interactive Care (ICIC) System for Care of the Elderly: A Survey-Questionnaire Study
Source: PLoS One. 2012 Aug 1;7(8):e40591. doi: 10.1371/journal.pone.0040591 (PMC3411612; doi:10.1371/journal.pone.0040591)
Supplement: Table S4 — Distribution TAM-2 scores for Intelligent Watch (n = 29). (DOC) [file pone.0040591.s004.doc]

| **Table S4.** Distribution TAM-2 scores for *Intelligent Watch* (n=29) | | | | | | | | |
| --- | --- | --- | --- | --- | --- | --- | --- | --- |
| Item | Distribution in percentage (%) | | | | | | | Mean average |
|  | Excellent | Good | | Fair | | Poor | |  |
|  | 7 | 6 | 5 | 4 | 3 | 2 | 1 |  |
| 1. If I got a chance, I would use “Intelligent watch.” | 66.7 | 20 | 3.3 | 10 | 0 | 0 | 0 | 6.4±1.0 |
| 2. If gave me “Intelligent watch,” I would definitely use it. | 70 | 16.7 | 6.7 | 6.7 | 0 | 0 | 0 | 6.5±1.5 |
| 3. Using “Intelligent watch” improves the quality of taking care of me. | 55.2 | 10.3 | 24.1 | 6.9 | 3.4 | 0 | 0 | 6.07±1.19 |
| 4. Using “Intelligent watch” enhances my ability on taking care of me. | 56.7 | 20 | 16.7 | 6.7 | 0 | 0 | 0 | 6.3±1.0 |
| 5. Using “Intelligent watch” saves me time, and makes it easier to take care of me. | 63.3 | 13.3 | 16.7 | 6.7 | 0 | 0 | 0 | 6.3±1.0 |
| 6. Using “Intelligent watch” improves my health. | 50 | 20 | 20 | 6.7 | 3.3 | 0 | 0 | 6.1±1.1 |
| 7. My interaction with “Intelligent watch” is easy for me to understand. | 36.7 | 13.3 | 26.7 | 23.3 | 0 | 0 | 0 | 5.6±1.2 |
| 8. I find it is easy to learn using “Intelligent watch.” | 48.3 | 31 | 13.8 | 6.9 | 0 | 0 | 0 | 6.2±0.9 |
| 9. Overall, I find the “Intelligent watch” easy to use. | 51.7 | 31 | 13.8 | 3.4 | 0 | 0 | 0 | 6.3±0.9 |
| 10. I find it easy to get “Intelligent watch” to do what I want it to do. | 64.3 | 14.3 | 17.9 | 3.6 | 0 | 0 | 0 | 6.4±0.9 |
| 11. Overall, I am satisfied with the quality of “Intelligent watch.” | 24.1 | 24.1 | 31 | 10.3 | 6.9 | 0 | 3.4 | 5.3±1.5 |
| 12. I have no doubt about the quality of “Intelligent watch.” | 21.4 | 25 | 32 | 14.3 | 3.6 | 0 | 3.6 | 5.3±1.4 |
| 13. I am glad to share the benefits of “Intelligent watch” with others. | 69 | 24.1 | 6.9 | 0 | 0 | 0 | 0 | 6.6±0.6 |
| 14. I will exchange the experience of using “Intelligent watch” with others. | 56.7 | 26.7 | 13.3 | 3.3 | 0 | 0 | 0 | 6.4±0.9 |
| 15. Obviously, using “Intelligent watch” helps me take care of me. | 56.7 | 20 | 16.7 | 6.7 | 0 | 0 | 0 | 6.3±1.0 |
| 16. I find it hard to distinguish between advantages and disadvantages. | 27.6 | 31 | 31 | 10.3 | 0 | 0 | 0 | 5.8±1.0 |
